# OmicLoupe: facilitating biological discovery by interactive exploration of multiple omic datasets and statistical comparisons

**DOI:** 10.1186/s12859-021-04043-5

**Published:** 2021-03-04

**Authors:** Jakob Willforss, Valentina Siino, Fredrik Levander

**Affiliations:** 1 Department of Immunotechnology, Lund University, Lund, Sweden; 2 Science for Life Laboratory, National Bioinformatics Infrastructure Sweden (NBIS), Lund University, Lund, Sweden

**Keywords:** Visualization, RShiny, Multiomics, Interactive, Explorative analysis

## Abstract

**Background:**

Visual exploration of gene product behavior across multiple omic datasets can pinpoint technical limitations in data and reveal biological trends. Still, such exploration is challenging as there is a need for visualizations that are tailored for the purpose.

**Results:**

The OmicLoupe software was developed to facilitate visual data exploration and provides more than 15 interactive cross-dataset visualizations for omics data. It expands visualizations to multiple datasets for quality control, statistical comparisons and overlap and correlation analyses, while allowing for rapid inspection and downloading of selected features. The usage of OmicLoupe is demonstrated in three different studies, where it allowed for detection of both technical data limitations and biological trends across different omic layers. An example is an analysis of SARS-CoV-2 infection based on two previously published studies, where OmicLoupe facilitated the identification of gene products with consistent expression changes across datasets at both the transcript and protein levels.

**Conclusions:**

OmicLoupe provides fast exploration of omics data with tailored visualizations for comparisons within and across data layers. The interactive visualizations are highly informative and are expected to be useful in various analyses of both newly generated and previously published data. OmicLoupe is available at quantitativeproteomics.org/omicloupe

## Background

Omic analysis carries the potential to reveal new biological understanding and serve as a source of biomarkers. Still, omic data are challenging to work with, in part as they often contain considerable variation within and between experiments driven by both biological and technical factors, such as differing experimental conditions or sampling procedures. This variation needs to be considered to correctly interpreting the data. Furthermore, choices of algorithms and statistical procedures for processing the data cause additional differences in the final results [1, 2]. The variation seen in the data can represent valuable biological trends, but can also be caused by nuisance factors, such as batch effects [3] or sample-to-sample technical variation. If the sources of trends in a dataset are understood, the dataset’s reliability can be assessed, and robust approaches of analysis and follow-up studies can be designed. Visualization is a critical tool for developing this understanding.

In comparative studies, one commonly overlooked aspect is the in-depth analysis of how individual features, such as transcripts or proteins, detected in one set of samples behave in other samples, datasets, or types of omics. Quality visualizations such as principal component analysis (PCA), and visualizations based on the outcome of statistical comparisons such as volcano plots and p-value histograms are often used to study trends within datasets. As an extension, several approaches to multiomics have been presented where the aim is to project down multiple sets of data to the same low dimensional space, such that they can be jointly visualized and inspected [4–6]. These provide useful overviews of multiomic datasets, but does not offer a detailed view of how individual features behave across multiple datasets in statistical comparisons.

As visualization is an important tool to fully explore omics datasets and to highlight features that can be difficult to assess with numbers alone, there are several new software solutions for omic data visualization presented over the past few years. These include a range of user-friendly stand-alone software for omics visualization such as Perseus [7] for proteomics, or shiny-based software such as ShinyOmics [8], which provides a flexible quality-oriented interface to omic data, and WIlsON [9] providing high-quality interactive figures based on an open file format but only limited abilities to compare features. Intervene is a software focusing on comparisons [10], aiming to provide various types of overlap information, but only based on fold change information and not allowing for feature-by-feature examination. Furthermore, software solutions dedicated to incorporating multiple layers of omics such as MixOmics [11] have extensive multiomics integration capabilities, but does so on a sample-wide scale rather than focusing on the behavior of single features.

Here, we propose an approach where single dataset visualization approaches are expanded to allow direct comparisons across datasets. Use cases are, for example, (1) Biomarker studies where an initial set of candidates is to be validated (2) Time-series experiment where the global expression is inspected, for instance, at different times after infection (3) Multiomics experiments where multiple types of data are produced for the same or similar biological systems and (4) Detailed studies of comparisons between methods or software approaches. To facilitate such analyses, we here introduce the interactive software OmicLoupe, which leverages additions to standard visualizations to allow for explorations of features and conditions across datasets beyond simple thresholds, giving insight which otherwise might be lost. The tool aims to be easy to use, directly interface with upstream software and to enable exploration and exporting parts of particular interest in the data. In the present work, we further demonstrate how OmicLoupe can be used to rapidly explore complex datasets in three different use cases.

## Results

### Software implementation

To improve the accessibility and capability of analysis of complex datasets, we developed OmicLoupe. It is an interactive piece of software accessible through any web browser, which can either be accessed online or installed and launched locally as an R package. A Singularity container for execution without any prior dependency installation, and video tutorials are available to increase its accessibility. The code follows a modular design, promoting the extension of OmicLoupe with additional visualizations in the future.

**Fig. 1:**
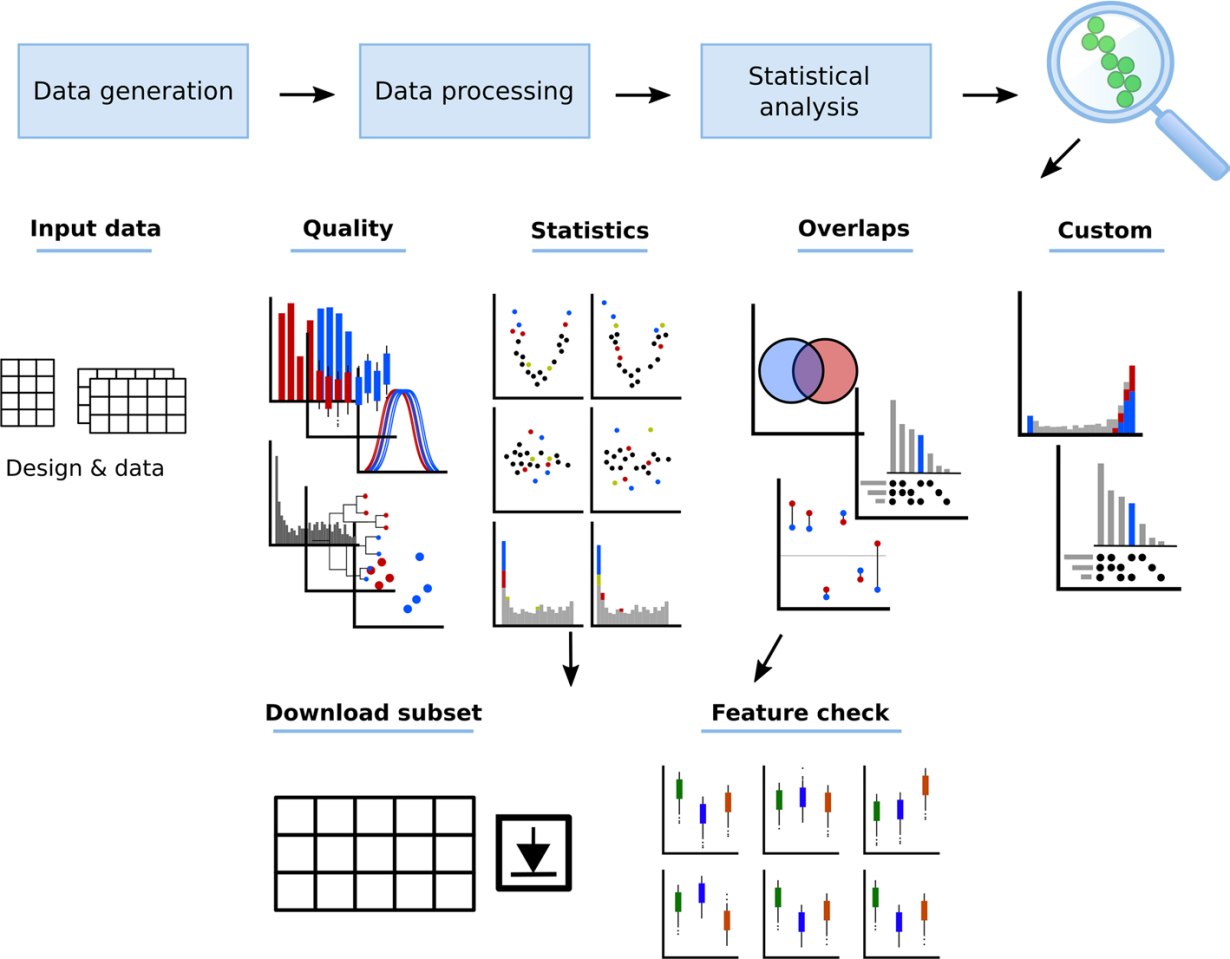
The OmicLoupe workflow. OmicLoupe is designed to easily interface with the upstream data generation process and to work on any expression data matrix. It provides the ability to explore up to two datasets, and provides comparative views between statistical contrasts performed either within one dataset or across multiple. It is organized in modules allowing rapidly shifting from a sample-wide view, to inspect individual statistical comparisons, overlaps between multiple comparisons, to understanding single features (Adapted from schematics shown at OmicLoupe’s home page [36])

OmicLoupe is built as a collection of modules, each performing a certain part of the analysis (Fig. 1). It is built to fit into upstream workflows and can handle any combination of one or two expression datasets where the data are presented as tables with samples as columns and features (genes, proteins, transcripts or other measured features) per rows (illustrated in Additional file 1: Materials S1). If visualizing two datasets, one column needs to contain shared IDs such as gene IDs to map the two datasets. If multiple entries map to the same ID, for instance in the case of multiple transcripts mapping to one gene ID, OmicLoupe can still combine these datasets by using the first listed entry for each ID. Alternatively, the user may preprocess the data such that the IDs are unique. Statistical visualizations require columns with p-value, false discovery rate (FDR) corrected p-values, fold change (difference of means between the two compared groups) and average feature level. These values are provided by up-stream software such as NormalyzerDE [12] or R packages such as Limma [13] for most types of omics or DESeq2 [14], for RNA-seq expression data. After loading the data in the web interface, the visualizations can be accessed immediately.

The general analysis workflow is shown in Fig. 1. The workflow starts with the user assessing their data using the sample-wide quality visualizations, including boxplots, density plots, bar plots, dendrograms, histograms, and principal component plots. These visualizations commonly reveal outlier samples and the presence of systematic effects in the data. Further, for studies involving two datasets, OmicLoupe provides the side-by-side study of whether these effects are uniquely present in one or both of the datasets. These visualizations help the user to make decisions on how to best perform analysis such as outlier omissions, or decisions on what statistical comparisons to perform, and to judge the reliability of the data.

Next, the user can screen overlaps between statistical comparisons and sample conditions by inspecting whether features pass specific statistical cutoffs (*p* value, FDR, optionally in combination with fold change) in one or several statistical comparisons (i.e. specific treatments or time points) or datasets. This overlap is illustrated by Venn diagrams for pairwise comparisons and UpSet plots [15] for a higher number of comparisons, with the UpSet visualization designed to efficiently compare a high number of overlaps. Further, for statistical comparisons, overlaps can be split by fold direction giving a better sense of whether overlaps indicate a shared abundance pattern. A novel visualization illustrates the fraction of features that change abundance in the same direction for low- and high- p-values, and the fold patterns of shared features are highlighted. These illustrations jointly provide a detailed view of similarities between contrasts. For both statistical and qualitative UpSet plots and for the Venn comparisons, subsets can be directly inspected and exported. Single features can be chosen for closer inspection in the Feature check module to evaluate in detail how their abundance values are distributed over any sample condition, shown either as raw data points or by using box- or violin plots.

Finally, to explore patterns across comparisons, the user can study pairwise comparisons and inspect how either significant or selected features in one dataset distribute across the volcano and MA scatter plots, as well as in p-value histograms. These visualizations can further be colored on any user-specified feature column and can be colored on respective loadings in the principal component plots, revealing groups of features strongly linked to specific principal components. Together, this yields an in-depth understanding of similarities and differences between contrasts and can display target features of interest. Similarly, for overlaps, single features can be selected and inspected in the Feature check module.

**Fig. 2:**
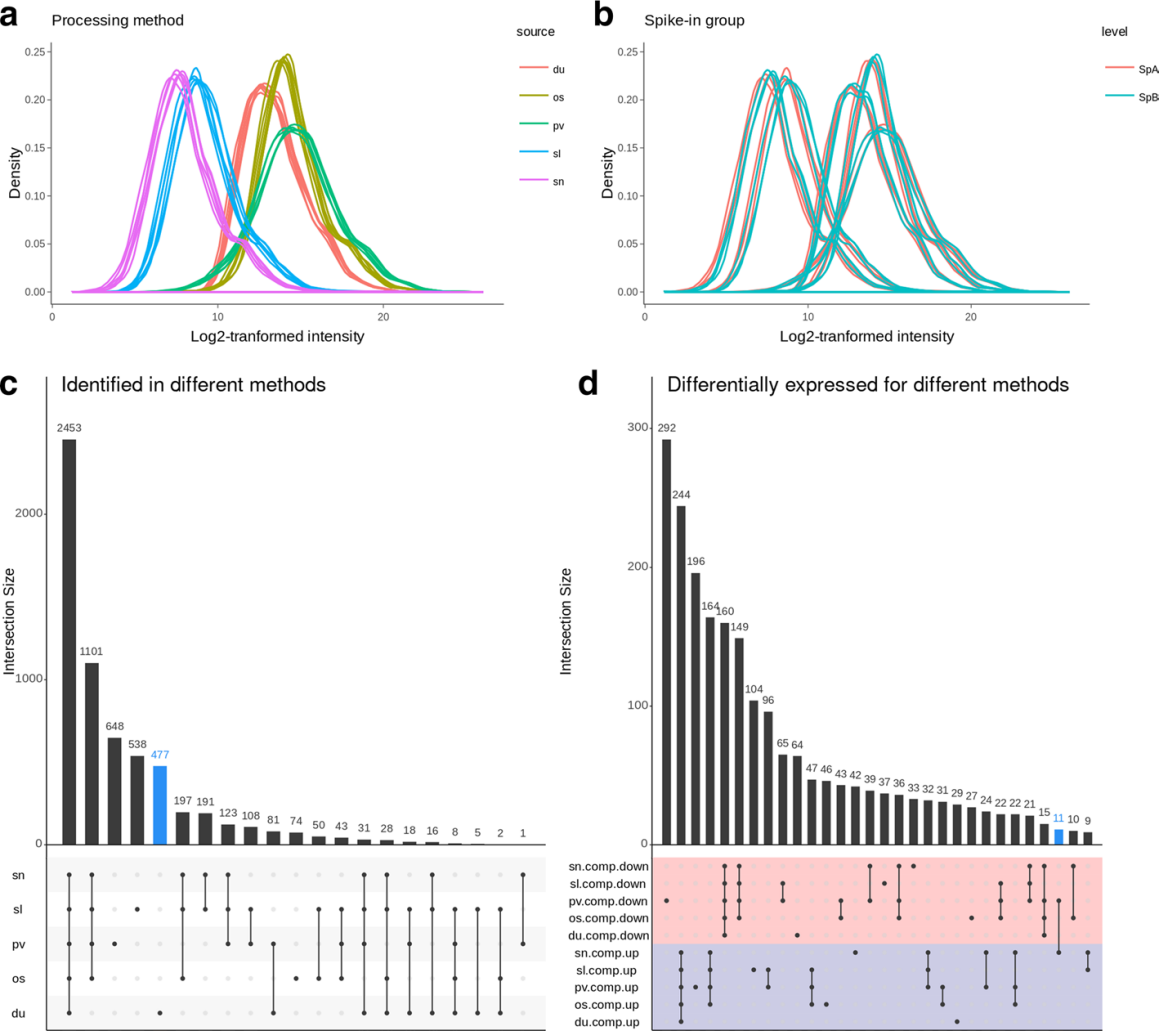
Effects of data processing using different software. Illustrations of data after processing with software: PeakView (pv), Skyline (sl), OpenSwath (os), DIA Umpire (du) and Spectronaut (sn). **a**, **b** Density distributions, available in OmicLoupe’s Quality panel, are colored on the data source (left) and spike-in level (right), respectively. **c** UpSet plot of features uniquely identified (not missing in all samples) after processing of different methods, with DIA Umpire highlighted. **d** UpSet plot of the features that were found as significantly differentially abundant (FDR < 0.05, log2 fold change > 1) when data had been processed using the different software. Features that are changing upwards or downwards in the comparison are displayed separately to visualize contradictory abundance changes due to differential processing. Eleven proteins that were deemed significantly changing, but with opposing direction of change after processing in PeakView and Spectronaut are highlighted in blue

**Fig. 3:**
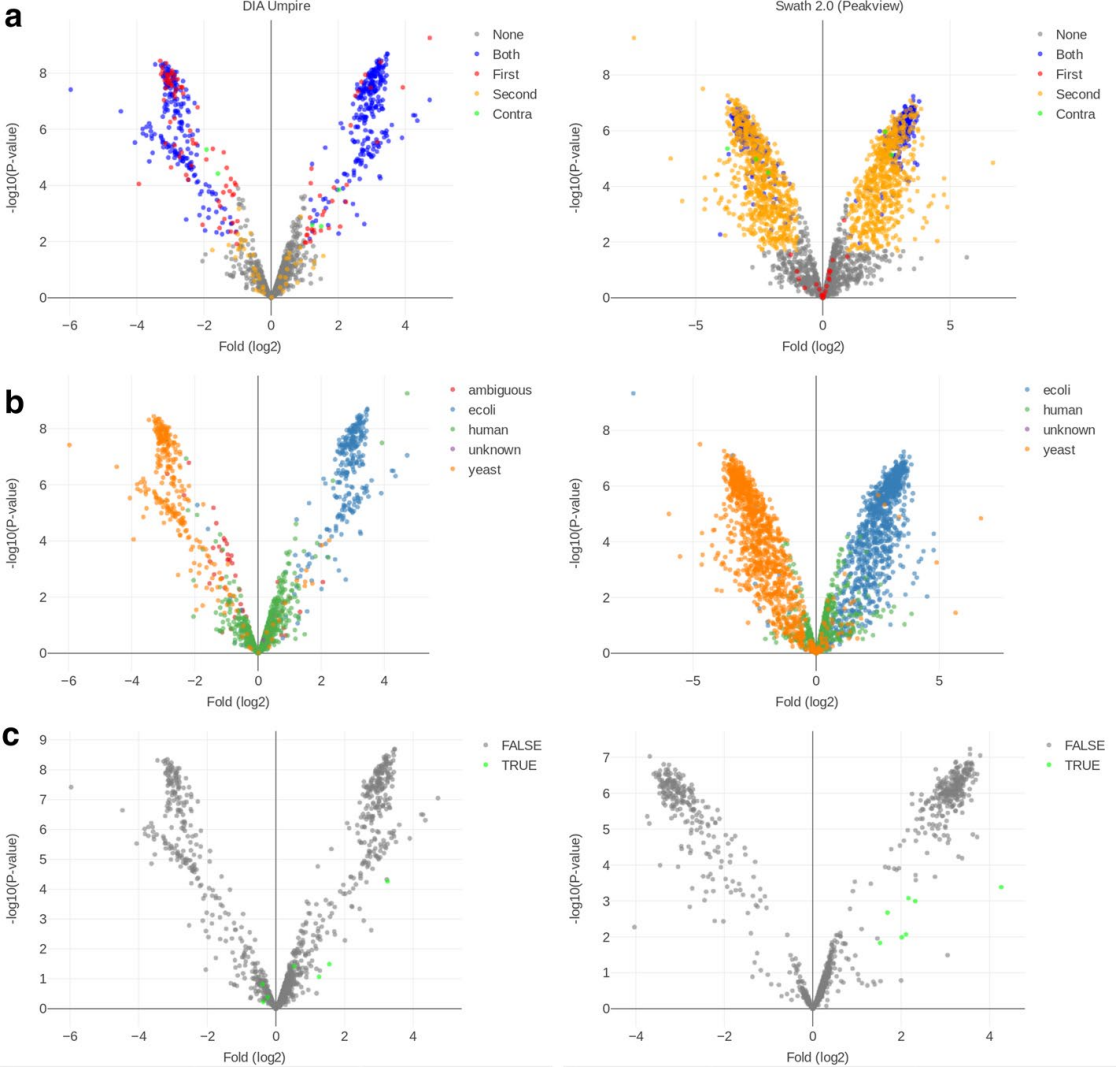
Comparison of contrast between spike-in levels in DIA Umpire and PeakView. These panels show part of the statistical interface in OmicLoupe, showing how the unadjusted *p* values for features relate to fold-changes in the statistical comparison between the two spike-in levels. **a** Features passing the significance threshold FDR < 0.05 and log2 fold > 0.5 in individual datasets, and in both. Green points (“contra” in the legend) are passing the significance threshold in both datasets, but with reversed log2 fold direction. **b** Coloring based on the spike-in source. **c** The outcome of interactively highlighting a set of six features only significant in PeakView and one significant in both. This reveals their distribution in DIA Umpire, showing that the features upregulated in both are true positives, while one of the two found in lower abundance in DIA Umpire is a false hit

**Fig. 4:**
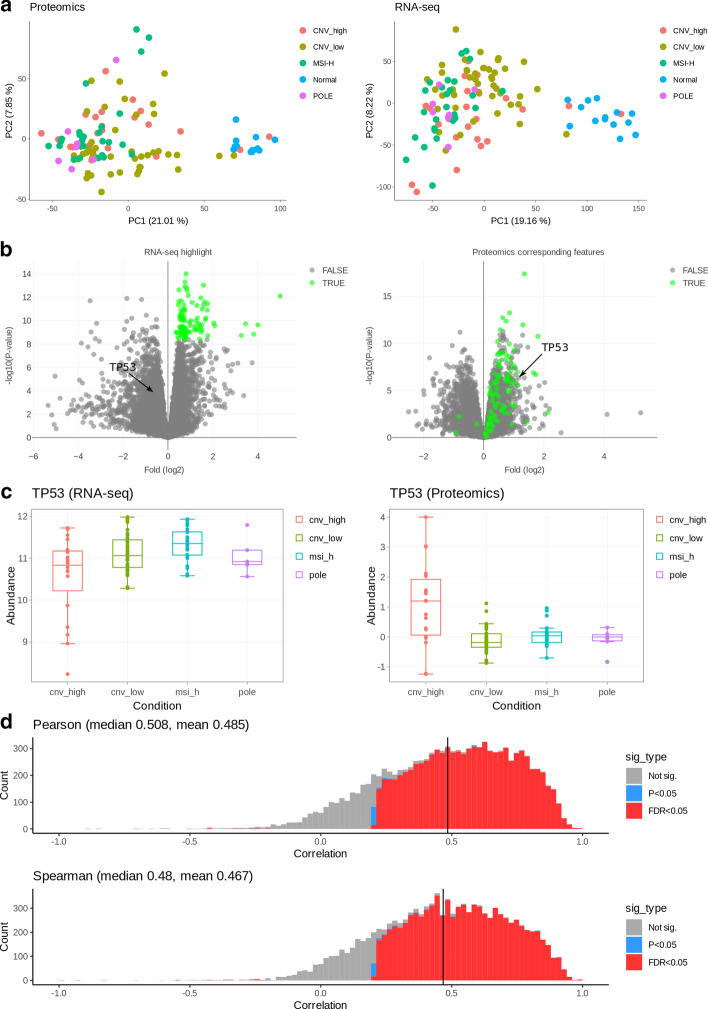
Illustrations of multiomics data investigated in Case 2. **a** Principal component illustration present in the quality module, comparing proteomics and transcriptomics. As can be seen, the major trends are similar between the two data types. **b** Distribution of how high-significance features upregulated in RNA-seq (left) distribute in the proteomics dataset (right). The positions of TP53 are indicated with an arrow. **c** Boxplots of TP53, identified in the dataset across the four studied sample classifications using the feature check module. Significant differences are present between CNV_high and CNV_low in both transcriptomics and proteomics. **d** Correlation distributions between the RNA-seq and proteomics features using the correlation module

**Fig. 5:**
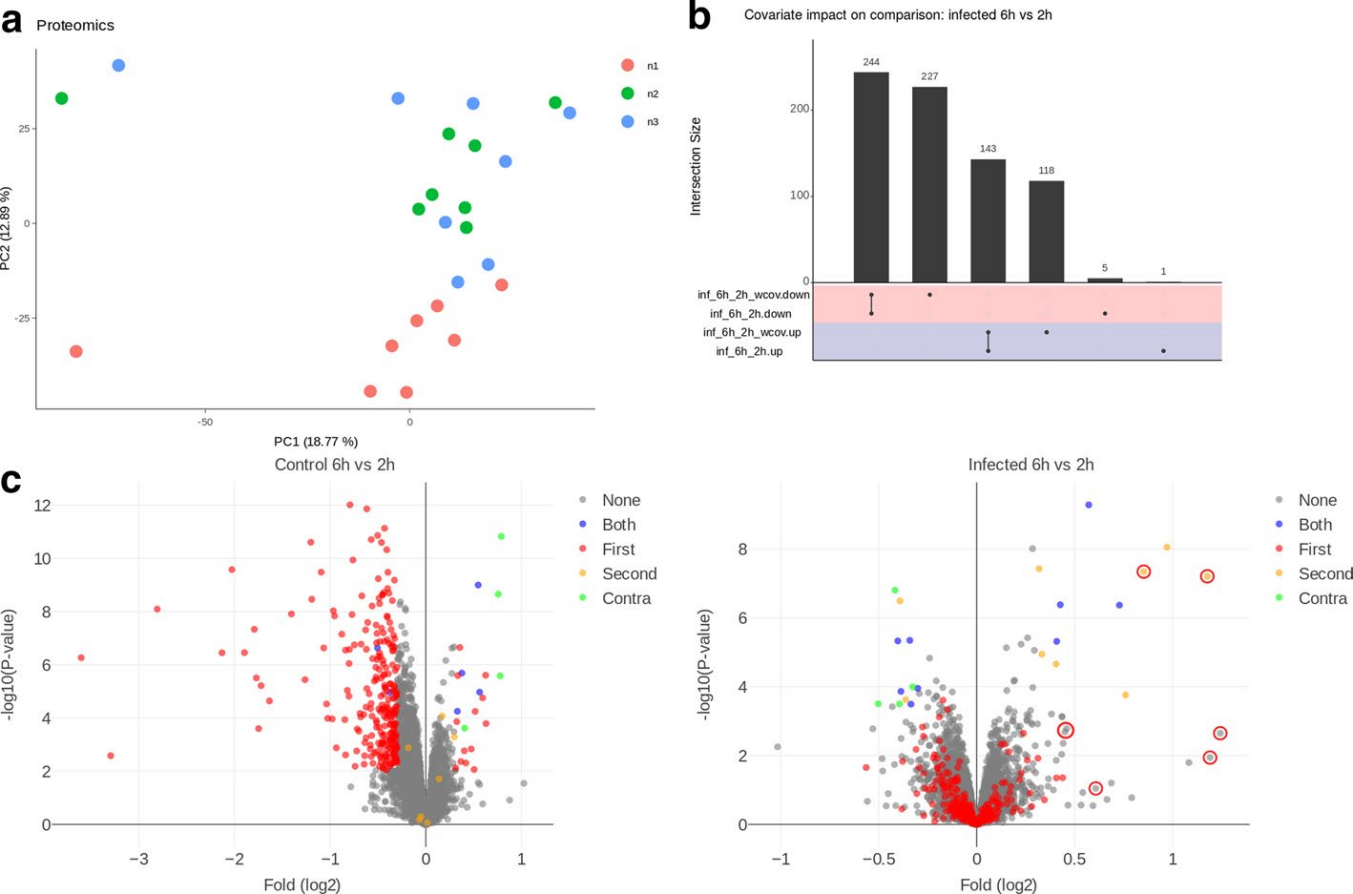
Quality inspection of the SARS-CoV-2 proteomics dataset. **a** Inspection revealed a separation of samples along the second principal component likely related to a plating effect. This was compensated for in subsequent statistical tests by including it as a covariate. Separation along the first axis is related to infection and time passed from infection. **b** The impact of performing differential expression analysis without and with inclusion of the putative plating number as a covariate. The inclusion of the covariate yielded 345 new statistical features while losing six as compared to not including the covariate. **c** Comparison of control samples 6 h and 2 h shows many features with a decrease in abundance, indicating that the mock treatment might influence the data. Comparison between infected samples at 6 h and 2 h show more limited differences, with seven detected viral proteins among those with increased abundance at 6 h indicated in red circles (log2 fold change > 0.3) out of which two passed the FDR threshold

**Fig. 6:**
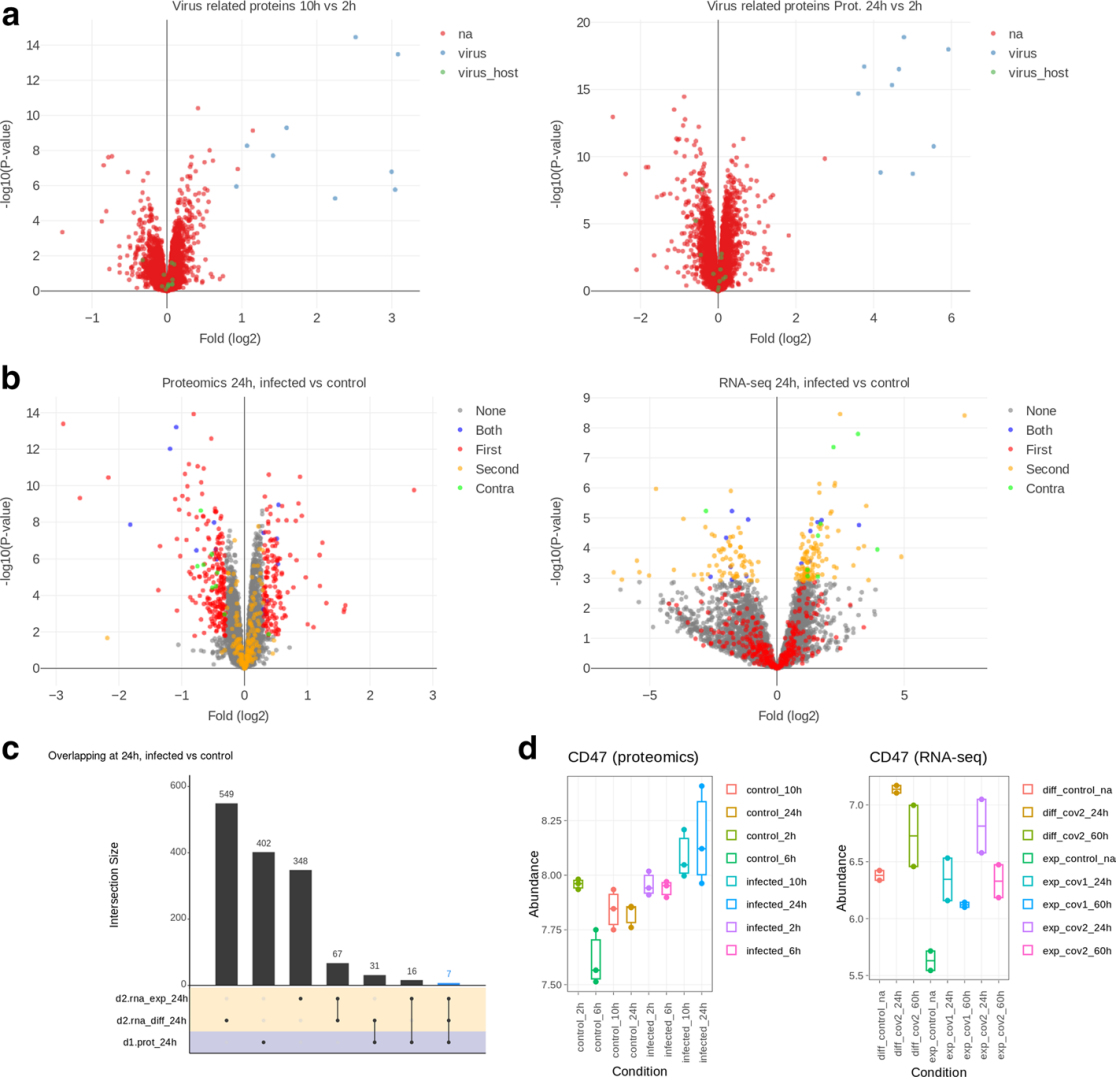
Inspection of trends in SARS-CoV-2 proteomics and transcriptomics datasets. Statistical analysis performed using the following settings: FDR < 0.05 and log2 fold change > 0.3. For both datasets, data from 24 h after viral infection are used **a** Inspection of infected samples, 10 h and 24 h compared to 2 h, colored by proteins known as virus proteins (‘virus’ in blue) and virus receptors (‘virus_host’ in green) revealed a clear upregulation among virus proteins. **b** Direct comparison of infected and control at 24 h after infection in proteomics and comparison between the proteomic dataset and the transcriptomic dataset (expansion media). The coloring is based on in which dataset the gene products pass the significance threshold. Green points (“contra” in the legend) are passing the significance threshold in both datasets, but with reversed log2 fold direction. This comparison revealed a set of shared proteins, both changing abundance in the same or opposite direction. **c** Illustration of the shared significant genes between proteomic (“prot”) and transcriptomic (differential “diff” and expansion “exp” media) datasets. **d** CD47 distribution at different time points in the proteomic study and in the transcriptomic data

Beyond the previously mentioned, further specialized visualizations are provided. An analysis approach for identifying features uniquely present in certain conditions is provided as an UpSet plot, which can highlight features for which the abundance is below detection limit for certain samples. A correlation plot allows direct illustration of feature correlation patterns between data layers based on the same set of samples, for instance multiomics or alternative software processing of the same dataset. All the plots discussed above can be downloaded in PNG or vector format and can be customized, providing publication ready visualizations (Figs. 2, 3, 4, 5 and 6 in the present study are examples of visualizations generated using OmicLoupe). Furthermore, to ensure reproducibility, settings used at any point can be saved as a JSON file (outlined in Additional file 1: Materials S2 and available at https://doi.org/10.5281/zenodo.4455520 for the figures presented in this work), and complete HTML reports including figures and settings can be generated for the different views.

To summarize, OmicLoupe provides a tool to rapidly assess datasets for technical trends and for in-depth studies of statistical comparisons and individual analytes. For this functionality, it provides several unique features, as illustrated in a comparison with other software in Table 1.

**Table 1:** Comparison of software for omics data visualization

Software	OmicLoupe	Perseus [7]	WIlsON [9]	ShinyOmics [8]	Intervene [10]
Web deployment	Yes	No	Yes	Yes	Yes
Open source	Yes	No	Yes	Yes	Yes
Platform independent installation	Yes	No	Yes	Yes	Yes
Container-based dependency management	Yes	No^1^	Yes	Yes	No
Compare multiple datasets	Yes	Yes	No	Yes	Yes (overlap)
Correlations between datasets	Yes	Yes	No	No	Yes
Statistical calculations^2^	No	Yes	No	No	No
Overview plots (Box-plot, PCA, dendrograms)	Yes	Yes	Yes	Yes	No
Network visualization	No	Yes	No	Yes	No
Side-by-side comparisons^3^	Yes	No	No	No	No
Single-feature visualization^4^	Yes	Yes	Yes	No	No
Feature selection from visuals^5^	Yes	No	No	Limited	No
Overlap visualizations^6^	Yes	Limited	No	No	Yes
Fold-oriented analysis^7^	Yes	No	No	No	No
Absence/presence comparisons	Yes	Yes	No	No	No
Export publication ready figures (vector format)	Yes	Yes	Yes	Yes	Yes
Export reports and settings	Yes	Yes	Yes	No	No
Tutorial videos	Yes	Yes	No	No	No

Simplified overview of features in a selection of software solutions for omics data visualization

^1^Installer provided

^2^“Yes” if calculation of statistical contrasts is performed interactively within the software, “No” if part of pre-processing is performed before loading data in the software

^3^Cross-comparison volcano plots or similar

^4^Detailed display of how individual measurements distribute over sample conditions

^5^Selection of subsets of features by direct interaction with the visuals. “Limited” if one type of visualization is provided, “Yes” for multiple types including interactive scatter-plots, and highlighting of subsections in overlap-plots with visualization of the selection

^6^“Limited” if only Venn. “Yes” if extended overlap representations such as UpSet plots

^7^Possibility to split visualizations to highlight features with similar or different abundance change directions in multiple comparisons

### Case 1: effects of data processing software on differential expression analysis outcome

To assess the utility and validity of the approaches introduced in OmicLoupe, we started by analyzing spike-in proteomic data that have previously been explored extensively in a comparison of data processing software for data-independent acquisition (DIA) LC–MS/MS data [1]. This dataset consists of *E. coli* and yeast proteins spiked at two different concentrations into a human proteome background. The two mixtures were analyzed in triplicates. In the original work, the data were processed using five different software, allowing for a comparison of their relative performance. The software used were PeakView (SWATH2.0, SCIEX), Skyline [16], OpenSwath [17], DIA Umpire [18], and Spectronaut [19], where only DIA Umpire was used without matching to a previously generated spectral library. This dataset was employed as an example with known ground truth where concentrations of proteins from different organisms were known, allowing assessment of how well the visualizations illustrate the known underlying trends. Further, it demonstrates how OmicLoupe can be used to assess the impact of different DIA software methods for processing the same set of samples.

Upon inspection in OmicLoupe, the quality control visualizations show that the choice of software impacts the absolute dynamic range of protein intensity, as illustrated in the density plots shown in Fig. 2a, b. Less obvious differences are seen between the spike-in levels, although upon inspection in a dendrogram, the difference between OpenSwath and PeakView appeared smaller than their respective spike-in levels (Additional file 1: Materials S3). It can be noted that the intensity values were scaled to reach similar levels in subsequent analyses in the original study for this reason. Qualitative inspection was performed to identify proteins only detected by certain software processing methods. Here, the majority of proteins (2453) were detected by all five methods, and 1101 proteins were detected by all methods except DIA Umpire. Conversely, DIA Umpire identified 477 proteins that were not detected by any other method (highlighted in blue, Fig. 2c), although PeakView identified a higher number of proteins uniquely (648, Fig. 2c). Upon statistically comparing the abundances between the spike-in levels, a considerable number of proteins were also uniquely identified as differentially expressed by PeakView (488 spike-ins, out of which 462 were correctly identified). Eleven proteins were found to be significant but with opposing direction of change when comparing PeakView and Spectronaut output (highlighted in blue, Fig. 2d). Out of these, all eleven were spiked-in yeast protein, correctly identified as downregulated by PeakView.

To further elucidate the underlying differences in the processed data from DIA Umpire and PeakView, a closer inspection of the statistical distributions was made in OmicLoupe. Out of the six statistical figures, the volcano plots are illustrated in Fig. 3 (all six can be found in the Additional file 1: Materials S4). Inspection of features passing the thresholds FDR < 0.05 and fold change (log2) > 1 (Fig. 3a) using the built-in side-by-side overlap coloring scheme shows a considerable number of common features with the same abundance change directions (blue) but it is notable that a larger number of features are identified only after PeakView processing, in most cases correctly so (95% of these were correctly identified spiked-in proteins, in line with the applied FDR-threshold of 0.05). These are distributed across all significance levels and folds, with no evident trends for a higher concentration of lower abundance values. A handful of features were, using the coloring scheme, found to be changing in opposite direction between the groups (green) and were compared to the expected regulation pattern. Here, it was found that all features with a positive log2 fold in both methods were true positives according to the type of spiked-in proteins, while out of the two with negative fold-change, one was a false positive. This indicates how OmicLoupe could be used to qualitatively give indications of the reliability of features across comparisons by using fold change. Interestingly, sets of features found to be changing in one group are clustering around zero-fold change in the other method, indicating a different ability of the software to handle these features. Further inspection of the ground truth (Fig. 3b) illustrates the possibility in OmicLoupe to change color scheme to highlight the respective types of spike-in. For PeakView, there seems to be, in particular for yeast, a considerable number of false negatives identified, while for DIA Umpire these are less common. To illustrate the joint use of the two methods, a subset of features identified after processing by both methods was inspected (Fig. 3c). Here, a set of features, only identified as differentially abundant after PeakView processing (yellow in Fig. 3a), was highlighted and their distribution after DIA Umpire processing inspected. One exception was significant in both cases, seen as the green point with lowest p-value (highest along the y-axis). Interestingly, the features represented a mix of true positives (*E. Coli*) and false positives (human). The true positives were found with greater fold change in DIA Umpire (two rightmost green points in Fig. 3C, DIA Umpire panel), which is closer to but still below the fold-change expected from the spiked-in concentrations. From these observations, we conclude that OmicLoupe allows for fine-grained analysis of differences resulting from data processing using different software and allows careful inspection of specific data points across multiple datasets.

### Case 2: analysis of matched proteome and transcriptome data

Multiomics studies of the same biological samples are becoming increasingly more frequent, but how to integrate the data types and finding important features remains challenging. We thus investigated how OmicLoupe can be used for direct comparisons of different data types taken from the same set of samples, to reveal features only detected in certain conditions, and common patterns of observed abundance level changes. For this purpose, a comprehensive multiomics dataset from endometrial cancer samples was downloaded [20]. Multiple types of data, including proteomics and RNA-seq, were acquired for the samples in the original study, and the features had been mapped to common gene identifiers. The samples are classified in different genomic sub-types, including Copy Number Variation (CNV) high, which includes serous and aggressive endometrioid cancers and CNV low, consisting of less aggressive endometrioid cancers. We focused our statistical analysis on a comparison between these two groups, as the differences between these subtypes were not extensively discussed in the original study.

A first view using the PCA module revealed a primary separation between most normal samples and tumor samples (Fig. 4a). This separation was similar in both proteomics and RNA-seq datasets, with few noticeable differences. PCA analysis without the control samples group was also performed using the function available in OmicLoupe (Additional file 1: Materials S5). Here, a partial separation between CNV high and CNV low is evident along the second principal component. To study the similarity of the statistical comparisons across the two data types, features with positive abundance change and with low p-values were highlighted in the RNA-seq contrast (by dragging directly in the figure) between CNV high and CNV low to see how these distribute in the corresponding contrast in the proteomics dataset (Fig. 4b). The majority of features were also upregulated in the proteomics data with three exceptions, namely FOLR3, STEAP1B and TBL1D31, which showed opposite direction of change in the proteome data. Another cancer gene of interest, TP53, which was discussed in light of TP53 gene mutation effects on the p53 protein level in the original study, was inspected using the feature check feature in OmicLoupe (Fig. 4c) and showed a seemingly reversed pattern between the transcript and protein levels for the compared groups. Finally, the overall correlation between transcriptomics and proteomics was studied. Pearson and Spearman correlations are illustrated in Fig. 4d and showed similar median values (Pearson 0.51, Spearman 0.48) to those presented in the original study (Spearman 0.48), with a small number of inversely correlated features. As suggested by the feature check of TP53, the transcript and protein showed little correlation for this gene (Pearson 0.10, Spearman 0.07), highlighting either a complex regulation pattern and / or possible quantification artefacts caused by gene mutations for RNA seq or proteomics, as well as the added value of explorations across multiple omic layers.

This demonstrates how OmicLoupe can be used to inspect similarities and differences between layers of omic data generated from the same set of samples, providing an improved understanding of both the general expression profiles and individual gene products.

### Case 3: cross dataset comparisons of proteome and transcriptome data from two published SARS-CoV-2 studies

When studying proteins, for instance involved in certain diseases, validation of key proteins in multiple experimental setups can provide valuable biological information. However, it can be demanding to handle omics data from different studies. We aimed to assess whether OmicLoupe could facilitate this process and aid in finding shared key features. Herein, we examined SARS-CoV-2 infection of two model systems from different studies with time series data to find overlapping features. The first study employed human colon epithelial carcinoma cell line (Caco-2) infected by the virus at different time points (2, 6, 10 and 24 h), with corresponding control samples, and with both proteomics and translatomics read-out, a proteomics-technique for measuring currently translated proteins [21]. In our work, we focused on the proteomics data. In the second study [22], the transcriptomic analysis had been performed on human intestinal organoids, representing the human gut, infected with the virus at time points 0, 24 and 60 h and in two different media: differentiation and expansion media. The datasets were first overviewed individually using OmicLoupe and then analyzed jointly to find common patterns.

For the proteomics study, the initial quality control revealed two aspects in the data influencing the subsequent analysis. Quality control visualizations using PCA and dendrograms revealed clustering of samples according to a sample name-based categorization, thought to be the plating numbers of the cell lines (PCA plot shown in Fig. 5a, dendrogram shown in Additional file 1: Materials S6). To compensate for this effect, this number was included as a covariate in the statistical tests, and the impact of including it was investigated in OmicLoupe. The inclusion of this covariate led to a considerably higher number of features detected as significantly different at the thresholds employed (FDR < 0.05 and log2 fold change > 0.3), as exemplified for the comparison of infected samples at 6 h versus infected samples at 2 h (Fig. 5b). Next, OmicLoupe was used to study control and infected samples independently (as illustrated in Additional file 1: Materials S7). Here, a clear pattern was seen in the infected samples, with the 24 h infected samples separating out along PC1, while the 10 h samples showed a weaker separation. In the control samples, the trend was less clear, and the control 6 h samples appeared as weak outliers. In order to study the potential impact of these group comparisons, control and infected samples at 6 and 2 h were compared, as depicted in Fig. 5c. A strong effect of decreasing abundance is seen in control 6 h, while in infected 6 h the trend is smaller, with known viral proteins being clearly upregulated. We verified that this was not due to overall imbalances in the dataset by inspection in OmicLoupe using a boxplot illustrating the log2-transformed intensities of each sample (shown in Additional file 1: Materials S8). This unexpected distribution of the 6 h control samples led us to focus on comparisons between infected samples, and the 24 h infected versus control comparison.

To study the viral distribution between the infected conditions, we highlighted proteins with known annotation related to either virus or other proteins thought to be related to SARS-CoV-2, such as virus receptor proteins [23] in comparisons of 6, 10 and 24 h infected samples compared to 2 h infected samples. Figure 6a clearly shows how the viral proteins are increasing in abundance in infected cells at 10 h and even more at 24 h. This first overview serves as quality control and is in line with what was shown in the original study. For the transcriptomic dataset, using the same cutoff parameters used for the proteomic study, a first evaluation shows that at 24 h after infection in the differentiation media, 654 transcripts are differentially expressed, while in the expansion media after 24 h of infection, 438 transcripts are differentially expressed.

Furthermore, to study the potential of OmicLoupe, the results from the proteomic study were compared to the transcriptomic dataset. Here, the two datasets can easily be uploaded and compared based on their time points. To make a similar comparison in the two datasets, we decided to compare the proteomic and the transcriptomic data at 24 h, despite one outlier sample being identified in the transcriptomics data at this time point (the PCA plot for the transcriptomics data illustrating this outlier is shown in Additional file 1: Materials S9). The distribution of the significant genes in the proteomic study in comparison with the transcriptomic study (expansion media), is depicted in the volcano plot in Fig. 6b. Of particular interest are the significant genes that are shared between the datasets at 24 h after infection. At the set threshold (FDR < 0.05 and log2 fold change > 0.3), 38 differentially expressed genes are shared between the proteomic and the transcriptomic data after 24 h of infection in the differentiation media. For the extension media, 23 significant genes are shared between the proteomic and transcriptomic datasets after 24 h of viral infection (16 genes exclusively, and 7 genes also shared with the differentiation medium). The overlap between the IDs in both datasets is displayed in the UpSet plot in Fig. 6c. Interestingly, 7 genes were overlapping between the proteomic dataset and the transcriptomic study (differentiation and expansion media). As an example, one of those shared genes, CD47, is depicted in Fig. 6d. CD47 is a leukocyte surface antigen, which has been shown to be upregulated after a viral infection, including SARS-CoV-2 infection, as a host response to the infection [24]. These overlapping groups were further analyzed in STRING [25] to investigate the relevant pathways connected to these significant genes identified. Of biological interest is that one of the main regulated Reactome [26] pathways is neutrophil degranulation, which in many studies has been reported as a key biological process during the SARS-CoV-2 infection [27–29].

In summary, in this third case study, OmicLoupe was used to perform a parallel analysis of two datasets from different types of omics (proteomics and transcriptomics) to investigate the response to infection over time. Both these datasets were obtained from published studies. By straightforward visualizations, we demonstrate the feasibility of using this tool to easily identify significantly changing gene products, common to both datasets, which can be used for further analysis, such as GO enrichment and pathway analysis.

## Discussion

Despite the recent addition of several new visualization software, we saw a lack of functionality for exploring multiple comparisons across datasets, and developed OmicLoupe to address this. Key features in OmicLoupe like the side-by-side data distribution comparison volcano and MA plots, with coloring of features according to abundance change direction across the comparisons, do not exist in other solutions (See Table 1 for comparison with other software). Furthermore, the ability to rapidly switch to individual feature views across samples, enable a deeper understanding of the individual features in the data.

The implementation of UpSet plots with optional splitting based on changes in abundance direction, can rapidly help in determining reproducibility across datasets. While standard statistical comparison, using strict thresholds in many cases is the default option, underlying trends can be found in plots such as UpSet with less strict thresholds, when the data are lacking power.

Here, we explored three diverse datasets to highlight different aspects of OmicLoupe’s functionality. By comparing the impact of different proteomics software processing methods, we could study in detail differences in outcome between the methods, and identify specific features handled correctly only by one or some of the methods. Next, multiomics exploration with both transcriptomic and proteomic data obtained from the same samples gave the opportunity to explore features across omic layers. Here, we identified an overall similarity of trends across the omic data layers, and rapidly illustrated the correlations of transcripts and proteins. Further, we visualized key features in detail, including TP53, a key protein discussed in the original study, and detected differences at transcript and protein level. This demonstrates how OmicLoupe can confirm and provide extended knowledge for existing data. Finally, we used two separate SARS-CoV-2 studies to profile intestine cells during infection. OmicLoupe was used to identify and navigate technical limitations, including a batch effect, and a seeming lower reliability of one set of control samples. The overall regulation patterns were relatively different, as expected due to the different types of samples, but still subsets of features with joint abundance changes were identified. These were downloaded and enriched, revealing biological trends in line with what had been observed in prior studies.

The cases presented in this manuscript are common examples of challenges encountered when analyzing omics data. Beyond these, the OmicLoupe software has the potential to be used in a wide range of scenarios, to better understand both single- and multiple- omics datasets. To this end, we believe usability is of critical importance for this kind of software, and OmicLoupe has a straightforward interface, with user help text complemented with video tutorials at the website. Having these at hand may mean the difference in how extensively the data could be explored, and thus how well they can be understood. We thus encourage users to test the software, provide feedback about its functionality and to comment on possible useful new extensions.

## Conclusions

Here, we have presented OmicLoupe, which both introduces novel approaches for comparative visualization cross dataset and presents these in an interactive easy-to-use software. We have demonstrated its utility on three diverse datasets, starting with a technical dataset to demonstrate how OmicLoupe can be used for comparing processing methods and how the cross-comparison fold can provide important information. Secondly, we explored a multi-omics cancer dataset illustrating how same-sample cross-omics can be readily illustrated. Finally, to demonstrate its versatility, we reanalyzed two recently published SARS-CoV-2 datasets, performed comparative explorations of these datasets and rapidly identified proteins and RNA transcripts showing the same abundance change trends across both studies. Based on these results and usage on other datasets, we propose that OmicLoupe can be a versatile tool in many expression omics-based analyses, both for novice and expert users. We provide it for usage by the community, as an R-package and as an online server.

## Methods

### Development

OmicLoupe is implemented using R (v3.6.3) and Shiny (v1.4.0.2), using packages providing interactive visualizations: Plotly (v4.9.2.1), DT (v0.13) and packages for data visualization: ggplot2 (v3.3.0), GGally (v1.5.0), UpSetR (v1.4.0, [15] ) and dplyr (v0.8.5) for data processing. The code is developed in modules to facilitate reusability. Further, a Singularity container [30] was prepared allowing immediate local execution without being required to install the R package dependencies.

### Dataset analysis

OmicLoupe was evaluated using three datasets covering different use cases. An R notebook containing the code used for preprocessing the datasets together with an HTML-document with the code output is outlined in the Additional file 1: Materials S10 and accessible on the https://doi.org/10.5281/zenodo.4455520. For all principal component plots, entries with missing values were filtered and scaled prior to visualization internally in OmicLoupe. For dendrograms, entries were similarly filtered and clustered using the average distance between clusters as distance metric internally in OmicLoupe.

#### Case 1: Technical spike-in dataset

A technical dataset was employed where proteins from human, *E. coli* and yeast had been spiked in at controlled concentrations [1] and subsequently analyzed using five different DIA methods. The data was downloaded from ProteomeXchange [31] at the ID PXD002952, selecting the data generated on the TripleTOF 6600 instrument with 32 fixed-size windows for all five methods. The HYE110 dataset was used, from two levels composed of 67% w/w (weight for weight) human protein in each case and 3%/30% w/w *E.coli* and yeast reversed between the two conditions, yielding an expected spike-in difference of log2 fold 3.3. The raw data matrices were preprocessed both into five separate data matrices, and into a single merged matrix consisting of all joint protein entries. They were subsequently log2-transformed and rolled up to protein level using an R-reimplementation v0.9.3[32] of the DanteR RRollup [33], using default settings and excluding proteins supported by a single peptide, yielding the following number of proteins: DIA Umpire (du) 3119, OpenSwath (os) 3656, PeakView (pv) 4615, Skyline (sl) 4912, Spectronaut (sn) 4141. For each method, two sets of three replicates from each spike-in level were provided Statistical contrasts between the two concentration levels were subsequently calculated using Limma (v3.42.2) [13] as provided by NormalyzerDE (v1.5.4) [12], and resulting p-values adjusted for multiple hypothesis testing using the Benjamini–Hochberg procedure [34]. No filtering of the features was performed, and missing values were kept as missing throughout the analysis. For comparisons of datasets, a joint table was generated containing data from all the five comparisons and matched based on their shared protein IDs, with proteins missing from certain datasets assigned as missing values for these.

### Case 2: Multiomics dataset

A multiomics dataset from a study investigating 95 prospectively collected endometrial carcinoma tumors [20] divided into four histological groups, including copy number (CNV)-high (serous cancer—a rare aggressive variant, and cancers with more than 50% penetrance of the endometrial wall) and CNV-low (less than 50% penetrance of the endometrial wall). The data matrices and meta information were downloaded from the supplementary information of the original study. Samples omitted from the original study were similarly omitted, as specified in the matrix obtained from the original supplementary, and samples present in both the RNA-seq and the proteomics data were used, resulting in 109 samples. Further, upon inspection with OmicLoupe the set of normal samples was identified as a strong outgroup and omitted to avoid influence in the statistical procedure, resulting in a final 95 samples. The original dataset contains multiple layers of omic data, out of which the following two were used in the present study: proteomics and mRNA levels. For the proteomics matrix contained 10,999 proteins, statistical contrasts were calculated using Limma (v3.42.2) [13] in NormalyzerDE (v1.5.4) and Benjamini-Hochberg [34] corrected FDR values were calculated. Missing values were kept as missing throughout the proteomics analysis. For the transcriptomics matrices the data was provided as RSEM estimated counts originally provided for 28,057 transcripts. Missing values were replaced with zeroes in the data and lowly abundant transcripts were filtered, resulting in 19,110 transcripts. It was then transformed using Voom with quality weights [35] as provided by the Limma package. Subsequent statistics were also calculated using Limma [13]. Statistical contrasts were for both data types calculated between samples classified as CNV-high and CNV-low. The data provided by the original study had already been mapped uniquely to gene IDs, which were used to combine the two datasets within OmicLoupe, resulting in 7805 shared entries.

### Case 3: SARS-CoV-2 datasets

The first dataset analyzed in this case study is a recently published SARS-CoV-2 proteomic dataset [21], where human colon epithelial carcinoma cell line (Caco-2) was infected by SARS-CoV-2 and proteomic analyses were performed on samples at four time points (2, 6, 10, 24 h after infection), both for infected samples, and samples treated with a mock infection. Three replicates are provided for each condition, for a total of 24 samples. The proteomic data and metadata were generously provided by the authors in the supplementary materials of the study, with 6381 measured proteins present in the original data. Zero-values were replaced with NA and kept as missing throughout the analysis, and the protein abundance values were log2 transformed. No filtering was done prior to the statistical analysis. Statistical contrasts were calculated using Limma (v3.42.2) [13] in NormalyzerDE (v1.5.4) [12] and resulting p-values FDR-corrected using the Benjamini–Hochberg procedure [34]. Initially, statistical comparisons were made between infected and control samples at each of the four time points (2, 6, 10 and 24 h after infection). After initial explorations in OmicLoupe a batch effect was identified, which was subsequently included as a covariate in the statistical test, as described in the results section.

The second dataset was from human intestinal organoids infected with SARS-CoV-2 in both differentiation and expansion media and analyzed at two time points after infection (24 and 60 h) using transcriptomics [22], with two replicates in each condition. The data was retrieved from the NCBI Gene Expression Omnibus (GEO) database, from the accession number GSE149312. The original dataset contained 18,014 transcripts, with no transcripts filtered prior to the statistical analysis. The data were TMM normalized with missing values as zeroes, and Voom transformed [35] with quality weights as provided by the Limma package. Subsequently, statistical calculations were carried out using Limma [13], comparing infected samples at 24 and 60 h after infection to the uninfected reference. P-values were FDR-corrected using the Benjamini–Hochberg procedure [34]. In both datasets, gene names were provided in one column. These were used in OmicLoupe to map proteins and transcripts between the datasets. In cases with duplicate entries being present, the first found entry was used (“Discard dups.” option in OmicLoupe), resulting in 5428 shared entries.

### Availability and requirements

The R package and its source code is available at github.com/ComputationalProteomics/OmicLoupe with https://doi.org/10.5281/zenodo.4455506

A public server running OmicLoupe, as well as links to video tutorials, can be accessed from the project home page.

A singularity container is available at singularity-hub.org/collections/4795 which allows execution with no need to install dependencies.

Project name: OmicLoupe.

Project home page: http://quantitativeproteomics.org/omicloupe

Operating system(s): Platform independent.

Programming language: R and Shiny.

Other requirements: none.

License: MIT license.

Any restrictions to use by non-academics: none.

## Supplementary Information

**Additional file 1**. Supplementary Materials S1–S10.

## Data Availability

The data used for case study 1 is accessible through the ProteomeXchange database under ID PXD002952 (http://proteomecentral.proteomexchange.org/cgi/GetDataset?ID=PXD002952). The data used for case study 2 is accessible from the study by Dou et al.[20] (https://doi.org/10.1016/j.cell.2020.01.026) and is based on the Supplementary Tables 1 and 2 provided with that article. The proteomics data used for case study 3 is accessible from the study by Klann et al.[21] (https://doi.org/10.1038/s41586-020-2332-7) and is based on the Supplementary Table 2 provided with that article. The transcriptomics data used for case study 3 is accessible through the Gene Expression Omnibus database under ID GSE149312 (https://www.ncbi.nlm.nih.gov/geo/query/acc.cgi?acc=GSE149312). The settings used in OmicLoupe to produce all visualizations in the manuscript and supplementary materials are available at https://doi.org/10.5281/zenodo.4455520 and outlined in Additional file 1: Materials S2. The R code for processing these datasets into a format compatible with OmicLoupe is available at https://doi.org/10.5281/zenodo.4455520 and outlined in Additional file 1: Materials S10. These output matrices are available from the corresponding author on reasonable request.

## Abbreviations

CNV: Copy number variation
DIA: Data independent acquisition
FDR: False discovery rate
LC–MS/MS: Liquid chromatography – tandem mass spectrometry
PCA: Principal component analysis
